# A Wireless Laser Displacement Sensor Node for Structural Health Monitoring

**DOI:** 10.3390/s131013204

**Published:** 2013-09-30

**Authors:** Hyo Seon Park, Jong Moon Kim, Se Woon Choi, Yousok Kim

**Affiliations:** 1 Department of Architectural Engineering, Yonsei University, 134 Shinchon-dong, Seoul 110-732, Korea; E-Mail: hspark@yonsei.ac.kr; 2 Center for Structural Health Care Technology in Buildings, Yonsei University, 134 Shinchon-dong, Seoul 110-732, Korea; E-Mail: watercloud@yonsei.ac.kr; 3 Dasumtek Incorporated, 222-7 Guro-dong, Guro-gu, Seoul 152-051, Korea; E-Mail: dstek@dstek.biz

**Keywords:** laser displacement sensor, wireless sensor node, structural health monitoring, displacement measurement, irregular building, mega-truss, under construction

## Abstract

This study describes a wireless laser displacement sensor node that measures displacement as a representative damage index for structural health monitoring (SHM). The proposed measurement system consists of a laser displacement sensor (LDS) and a customized wireless sensor node. Wireless communication is enabled by a sensor node that consists of a sensor module, a code division multiple access (CDMA) communication module, a processor, and a power module. An LDS with a long measurement distance is chosen to increase field applicability. For a wireless sensor node driven by a battery, we use a power control module with a low-power processor, which facilitates switching between the sleep and active modes, thus maximizing the power consumption efficiency during non-measurement and non-transfer periods. The CDMA mode is also used to overcome the limitation of communication distance, which is a challenge for wireless sensor networks and wireless communication. To evaluate the reliability and field applicability of the proposed wireless displacement measurement system, the system is tested onsite to obtain the required vertical displacement measurements during the construction of mega-trusses and an edge truss, which are the primary structural members in a large-scale irregular building currently under construction. The measurement values confirm the validity of the proposed wireless displacement measurement system and its potential for use in safety evaluations of structural elements.

## 1. Introduction

An accurate evaluation of damage to structures resulting from aging and the application of various loads throughout their life cycles can be used to evaluate the safety and serviceability of a building as well as to maintain and manage the structure. Structural health monitoring (SHM) based on sensor technology has been accepted as an important method for safety evaluations of engineering structures [1,2].

In SHM, the responses from a structure are measured using various sensors, and the measured data are transferred to a monitoring server using a wired or wireless transfer method for analysis and evaluation by administrators. The characteristics of the measured target structure and of various loads (wind load, earthquake load, and service load) determine the target elements, the response types, and the measurement sensors needed. Thus, the selection of a response index that can accurately assess damage to a structure is crucial, and conventional measurement devices such as accelerometers, strain gauges, and displacement transducers have been extensively applied for this purpose [3–6]. The accelerometer measurements are used to estimate damage by analyzing the changes in the dynamic characteristics of the structure [7]. However, in addition to structural factors, non-structural elements and environmental factors can significantly influence changes in the dynamic characteristics of a structure, which is a critical problem [8]. Although a strain gauge can accurately identify local damage, the installation of numerous strain gauges is required to evaluate the safety of all structural elements or the safety of an entire structure, a process that can prove challenging. Therefore, the direct measurement of displacement or deflection as a response index that can effectively represent damage to a structure is critical.

Although the measurement requirements for laboratory experiments can be easily controlled, various field characteristics should be considered during the selection and installation of measurement devices for onsite measurements of actual building displacements. In the case of contact-type sensors, *i.e.*, accelerometers and strain gauges, measurements are performed by direct contact with the target elements to be measured. In contrast, a conventional contact-type displacement transducer performs a relative displacement measurement from a reference frame located inside a building; these transducers are subjected to many limitations when installed at an actual site.

Due to these challenges and the importance of displacement measurements, applications of SHM using global positioning systems (GPSs) [9,10], laser Doppler vibrometers [11,12], terrestrial laser scanners [13,14], and vision-based methods [15,16] as well as existing displacement transducers have been proposed as potential methods of displacement measurement. However, these techniques cannot satisfy all of the requirements of cost effectiveness, accuracy, long-term measurement, and real-time monitoring when SHM is applied to real structures. Therefore, the development of a practical and economical displacement measurement system that is suitable for onsite application is needed.

The prevention of data loss and the development of a reliable transfer system are also crucial factors in SHM. A wired monitoring system, which was the foundation of early SHM, provides a relatively reliable transfer of measured data; however, the installation and maintenance of a wired network is costly [17]. The invasive effect of installing wired networks in a structure is also considered a major obstacle to the applicability of a wired SHM approach.

These problems associated with the structure of a wired network are directly caused by the cable-based transfer mode of measured data. To resolve this problem, a less costly wireless network system can be applied to real structures for measurement data transfer [18]. As a result, wireless networking has begun to attract increased attention in the field of SHM, and numerous studies have been conducted on wireless sensor development and network system construction [19]. However, major problems associated with the application of a wireless network system in the field include power consumption, time synchronization, multi-scale network topologies (scalability), decentralized data processing, and power-efficient data-driven usage strategies. These problems are directly related to the assessment of whether data can be reliably acquired by a wireless network on a long-term basis. Wired and wireless network systems for SHM applications each have specific strengths and shortcomings, resulting from the tradeoff between cost and reliability. Various studies and field application results indicate that wireless network systems are capable of effectively addressing these limitations in conjunction with the advancement of related technologies (e.g., sensors, wireless communication, and power-efficient technologies) and demonstrate great potential for progress.

In this study, we employed a laser displacement sensor (LDS) as a non-contact measurement device to directly measure the displacement of a structure and developed a wireless laser displacement measurement system using a customized wireless sensor node mounted with low-power devices to increase field applicability. Few cases exist in which a displacement transducer-based SHM has been applied to buildings. Cases in which wireless sensor nodes have been developed with respect to displacement sensors (*i.e.*, LDSs) are quite rare, although a customized wireless acceleration board and strain board have been proposed [20–22]. A characteristic of the proposed sensor node is that it uses low-power technology to obtain long-term reliable measurements, which are necessary for a wireless sensor network; the sensor and the sensor node switch to an active mode only during measurement or data transfer. The transfer-distance limitation can be overcome and data loss during transfer can be reduced using the code division multiple access (CDMA [23]) transfer mode as the wireless communication method. Site management can be conducted from a remote monitoring server using this long-distance transfer mode. To evaluate the reliability and field applicability in a large irregular building under construction, the proposed wireless laser displacement measurement system is applied to vertical displacement measurements occurring in the main structural elements (cantilever-type mega-trusses and an edge truss), and the validity of this system is evaluated based on the long-term measurement results.

## 2. Development of a Wireless Laser Displacement Measurement System

### 2.1. Laser Displacement Sensor

With respect to various onsite constraints associated with the installation of displacement transducers, non-contact displacement measurement devices exhibit greater applicability than contact-type displacement transducers. Therefore, an increasing demand exists for the use of these non-contact devices to measure the displacement of a structure for which the installation of devices is problematic. Certain examples of typical non-contact displacement transducers include eddy current sensors [24], capacitive sensors [25], confocal sensors [26], and optical sensors. The LLD-0100 model (JENOPTIK AG, Jena, Germany), with the ability to perform long-range measurements (0.2–35 m), is employed in this study. This long-range capability is the most basic requirement for onsite installation of a displacement transducer that can flexibly respond to the previously mentioned limitations and constraints. Although the accuracy of the proposed model, *i.e.*, 0.2 mm, is lower than the accuracy of other LDSs [27], a 0.2 mm accuracy is an acceptable error range because the measurement distance or the measurement range is the most important consideration at an actual site that contains numerous limitations for the installation of measurement devices *versus* a laboratory environment in which the measurement conditions are easily controlled.

The LDS employed in this study contains an optical mode that calculates distance by comparing the phase difference between a laser signal emitted from a sensor and the laser signal reflected from the measurement target. In addition to the ability to conduct long-range measurements, the LDS is rarely influenced by aspects of the external environment such as wind and temperature (operating temperature from −10 °C to approximately 50 °C), and it consumes little power. Another particular feature of the LDS employed in this study is its compatibility with a wide range of colors and materials of the reflector used to reflect the laser beam. Either the RS232 or the RS422 mode can be used as the data interface with this sensor. Either digital displacement or analog output can be selected as the data output format. The LDS also includes a trigger function that signals the start and finish of the measurement. The internal program uses an auto-trigger function, and the external trigger device uses a remote trigger function.

### 2.2. Wireless Sensor Node

**Figure 1.**
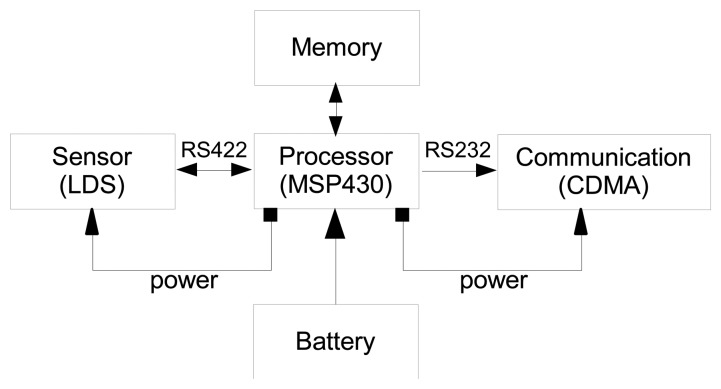
Wireless laser displacement sensor node.

**Figure 2.**
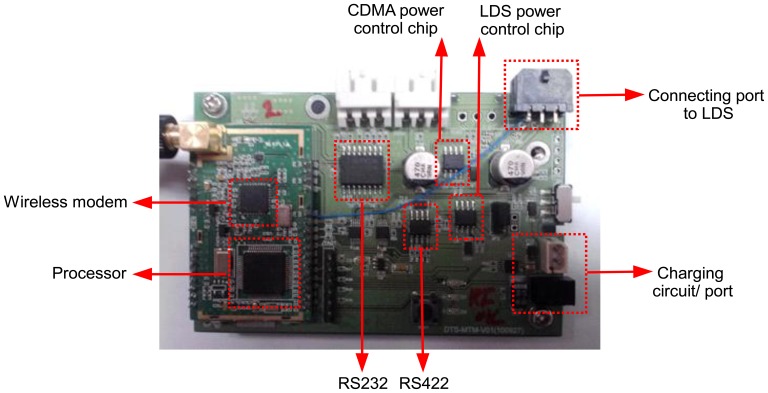
Wireless laser displacement sensor node.

The wireless sensor node developed in this study consists of a processor, a sensor module, a communication module, and a power supply module, as shown in Figure 1. Figure 2 illustrates the board of the wireless sensor node. The sensor module, communication module, power supply module, and processor are mounted on a single board. The size of the sensor node is designed with dimensions of 98 mm (width) × 69 mm (depth) × 34 mm (height) to meet the size requirement essential for field applications.

**Figure 3.**
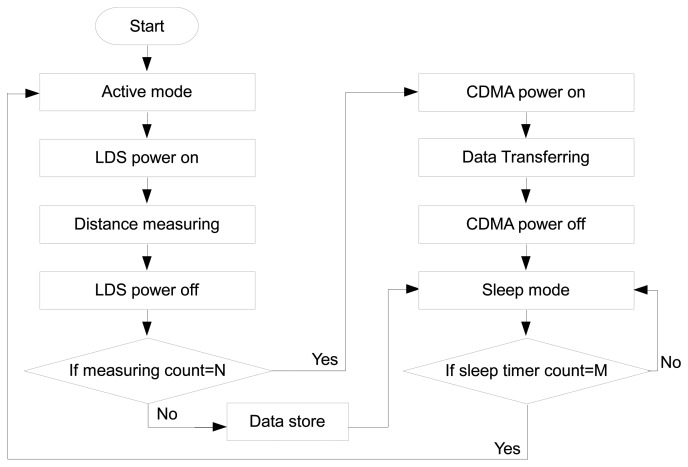
Operation of the wireless laser displacement measurement system.

The wireless sensor node is used to acquire and control the wireless transfer of measured data from the LDS. Its basic operating steps are illustrated in the flowchart in Figure 3, and the operating temperature of the proposed sensor node ranges from −35 °C to 80 °C. As depicted in the flowchart, the processor controls the power supply of the LDS through the sensor module, and the communication module controls the power supply of the CDMA module. In this study, the measurement and data transfer times are preset to enable control of the on/off mode of the power supply, which minimizes the power consumption of the limited battery capacity. In other words, once an instruction is delivered to begin the measurement of the LDS according to the preset schedule (M multiplied by the default time, where M is the preset number) and after a certain number of measurements (denoted by N in the Figure 3) have been obtained, the activation of the CDMA communication module is triggered for transfer of the stored measured data to the monitoring server.

#### 2.2.1. Processor

The processor controls the measurement start instructions, the acquisition of measurement information, and the transfer of measured data, which are all performed by the sensor node. In this study, an MSP430 microcontroller from Texas Instruments (Dallas, TX, USA) [28] is used as the processor in the sensor node to minimize power consumption. This processor is a flash-based unit that contains a 16-bit RISK architecture, 2-kB RAM, and a 256-byte flash memory.

The main control functions of the processor are performed by the power supply control module, the timer module, the laser drive module, and the CDMA module. The power supply control module controls the power supply by performing the on–off switching of the externally located power supply according to the preset schedule. The timer module calculates the time required to provide information with respect to the operating time of each module according to their respective preset schedules. The laser drive module controls the power supply of the laser module and obtains the measurement values via communication with the LDS. The CDMA communication module delivers the measured data to the remote server. This sensor node, which is capable of controlling both the measurement time and the transfer time, stores the measured information in the memory until it transfers the data at the preset transfer time. The most distinct feature of this processor is that the CPU contains five low-power idle modes [28] that can be selected by a user. A wake-up function is implemented by the digitally controlled oscillator (DCO) in 6 μs and changes the sleep mode of the CPU to the active mode of the CPU in low-power mode. All of the clocks [main clock (MCLK) and the sub-main clocks (SMCLK)] except for the auxiliary clock (ACLK) use the low-power mode to maintain the off state.

#### 2.2.2. Sensor Module

The sensor module serves as a sensor interface that acquires the data measured by the LDS. The RS422 mode is used for communication between the LDS and the sensor nodes. The LDS used in this study supports the serial interfaces of the RS232 and RS422 modes. Because the RS232 mode is susceptible to electromagnetic noise through the cables, the RS422 mode is selected for this study because it produces minimal interference and noise, even with the use of a long cable. This module is mounted on the board of the sensor node.

The information measured by the LDS is delivered to the sensor node as a type of digital displacement. The LDS employed in this study contains an A/D converter, which enables conversion of the analog data of the measured electric signal (voltage) into a digital signal and subsequent conversion into a physical displacement in the LDS. Therefore, the sensor module of the current sensor node is appropriately designed for the data output format (digital displacement) of the LDS. If other types of LDSs manufactured by other companies are required, the sensor module of the current sensor node should be modified accordingly. The existing sensor module relays the instructions for the start and finish of the operation in the LDS, the acquisition of the measured data, and the operation of the power supply to the sensor. The sensor module is always switched to the sleep mode to minimize power consumption, except during the time of measurement. This task can be accomplished by establishing a default time interval and counting the number of times (denoted by M in Figure 3) that the module is switched to active mode during a certain period.

#### 2.2.3. Wireless Communication Module

A short-range communication method using radio frequency (RF) waves is generally employed in a wireless sensor network (WSN). The RF waves are classified into low-frequency, high-frequency, ultrahigh-frequency, or extremely high-frequency bandwidths according to the applicable frequency band. An applicable onsite communication method should be selected depending on the field situation, the characteristics of the communication devices, the communication distance, and the amount of diffraction. In a WSN based on RF waves, a multi-hop method [29,30] is occasionally used due to the limitation posed by the transmission distance from each sensor node to the server. The CDMA mode (the communication method used in this study) is not limited by distance, and thus, the final transmission of the monitoring servers can be remotely located. Therefore, only onsite installations of the LDS and the wireless sensor node equipment are required, which increases the management efficiency of the measurement devices. The CDMA communication method uses a spread spectrum, and therefore, minimal data loss occurs during transmission. Unlike the wireless communication methods that use the radio-frequency (RF) signal band or ZigBee usually employed in wireless monitoring systems, CDMA communication exhibits good diffraction characteristics inside the structure, where various communication obstacles exist. In South Korea, where the target building is located, CDMA was officially selected as the standard for digital mobile phone systems in 1996, and this has successfully become commercialized worldwide. CDMA communication also consumes relatively little power and is thus considered appropriate for long-term wireless communication.

The majority of the power consumption in a wireless sensing unit occurs during wireless communication. Therefore, the data transmissions should be carefully controlled to achieve efficient power management; in other words, it is important to minimize power loss by controlling the amount of transferred data and the transfer time. In this study, the measured data transferred to the sensor node are stored in the sensor node and transmitted to a remote monitoring server only if the stored data exceed a preset threshold. This step constitutes a measure of the ability to provide a stable power supply, which has been considered as a critical problem for wireless sensing units that depend on a limited-capacity battery. Thus, if the transmission time is set in real time, real-time monitoring is also possible.

#### 2.2.4. Power-Saving Circuit

**Figure 4.**
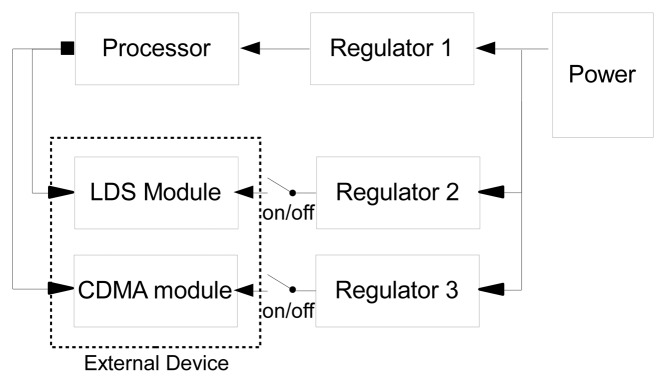
Power-saving circuit.

The power required for each module in the existing sensor node is supplied by a battery. The power required for the operation of the LDS is also supplied by a battery in the sensor node. Because the LDS measures distance by generating a laser beam, the power consumption is relatively high compared with the power consumption of general sensors. Therefore, power-saving technology is essential for performing long-term measurements. The basic principle of our power-saving device is to classify the power supplied to the sensor and the processor into measurement modes and non-measurement periods to minimize the power consumption during periods in which measurement and data transfer are not performed. To supply power to each module, a regulator is configured in terms of three different regulators, such that regulators 1, 2, and 3 are responsible for the power supply of the processor, sensor module, and CDMA communication module, respectively (Figure 4). Therefore, during the non-measurement period, the power supply of regulator 2 is blocked, whereas the power supply of regulator 3 is blocked during the non-transfer period, thereby increasing the efficiency of the power supply. Regulator 1 provides continuous power to the power supply so that the processor can facilitate control of the on/off modes of all modules according to the preset measurement and transfer programs.

Power consumption is measured during the sleep mode, the measurement period, and the transfer period to determine the power consumption of the entire system. The power consumption is approximately 840 μA for the sleep mode, approximately 123 mA for the measurement period, and approximately 50–200 mA for the CDMA communication period. These results indicate that the power consumption is considerably low during the sleep mode, whereas the majority of the battery power is consumed during the measurement and transfer periods. Therefore, calculation of the service life of the battery is based on the measurement period and the transfer period (denoted by N and M, respectively, in Figure 3). Determination of the measurement parameters is based on the measurement target, battery life, and mobile communication charge incurred by the CDMA, which enables suitable monitoring of various field situations via flexible management of the measurement and transfer periods. Although a power-saving mode is employed in the developed wireless sensor node, the problem of a stable power supply remains a critical issue when applied to long-term monitoring and locations that are inaccessible for battery change. Therefore, a further study is needed on this issue with respect to high-density energy storage technology and energy harvesting for wireless sensor nodes [31].

## 3. Target Structure (Building D)

**Figure 5.**
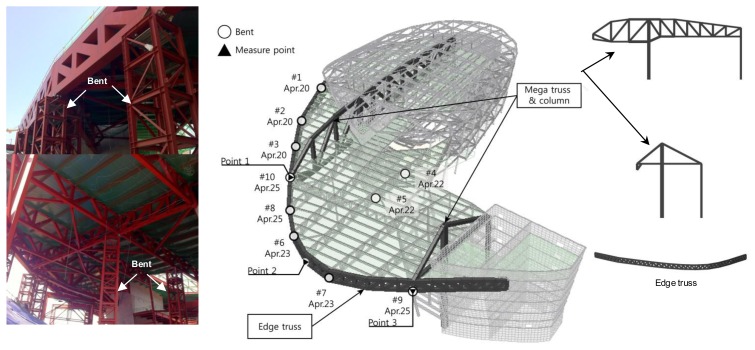
Target structure (building D).

The wireless LDS node in this study is employed for vertical displacement measurements of the main structural members of a building that is currently under construction (Figure 5). The target building (building D) is a large-scale irregular building designed to support an enormous space using large elements, *i.e.*, mega-columns and mega-trusses. Accurate construction of these main elements and the safety of the building under construction must be ensured during the construction phase of the building.

Because they are structural components of the building, the horizontal slabs that form the large spaces are supported by floor trusses; these floor trusses are connected to mega-trusses and edge trusses, with the mega-trusses connected to a mega-column to which the loads are ultimately transferred. In this structure, one end of the element in the mega-truss (which is supported by the mega-column) is of the cantilever type shown in Figure 5, and the two cantilever free ends of the mega-trusses are connected to the edge truss. Therefore, the vertical displacement at the free end of the mega-truss is an important measurement target for guaranteeing construction accuracy and structural safety during the construction and use of the building.

The building construction involves part-by-part fabrication of these large elements (mega-truss and edge truss), which are joined by welding. Each component of the edge truss is supported by the installation of a temporary bent (Figure 5), followed by joining of the components of the edge truss and connection of the edge truss to the mega-truss. Once the connection is completed, the temporary bents that were installed to support the edge truss and the cantilever free ends of the mega-trusses are removed. The vertical displacement at this time is the primary monitoring target and is the focus of this study. Here, we monitor sudden behavioral changes in the elements due to the removal of the bents by establishing a more frequent measurement and transfer interval once the bents are removed and by long-term monitoring using the proposed wireless laser displacement measurement system to measure the vertical displacement.

**Figure 6.**
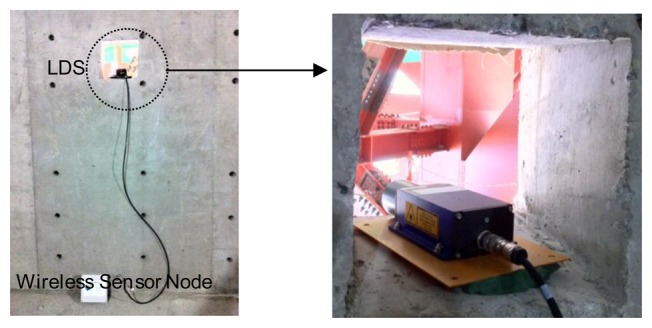
Wireless laser displacement measurement devices (LDS and wireless sensor node).

We use three measurement points [*i.e.*, two joint points between the mega-trusses and the edge truss and the center point of the edge truss (Figure 5)], where the largest deflection is expected to occur during removal of temporary bents, and perform the measurements by installing the LDS and the sensor node. It is best to measure the absolute vertical displacement of the members (mega-trusses and edge truss) by installing an LDS on the ground. However, due to the conditions of the construction site, such as the various construction equipment and movements, and because the measurement range of the LDS is limited (maximum of 35 m), it was impossible to install these sensors on the ground. Therefore, in this research, the LDSs were installed within the building in the areas least affected by the construction to measure the data in a stable manner (Figure 6). The LDS and the laser wireless sensor node are connected via cables for RS422 communication and are installed at the building site. The displacement data measured by the LDS are transferred to the wireless sensor node, and the measurement results are transferred to the remote monitoring server via CDMA communication.

The measurement results are integrated by the integrative management program installed on the monitoring server. The measurement results that are transferred to the monitoring server can be viewed in real time from an internet-enabled terminal, independent of time or space. Therefore, the field applicability is increased because all data transfers from the measurement stage to the data check stage are performed wirelessly by the administrator, with the exception of the short-wired component that connects the LDS and the sensor node.

## 4. Measurement Results

**Figure 7.**
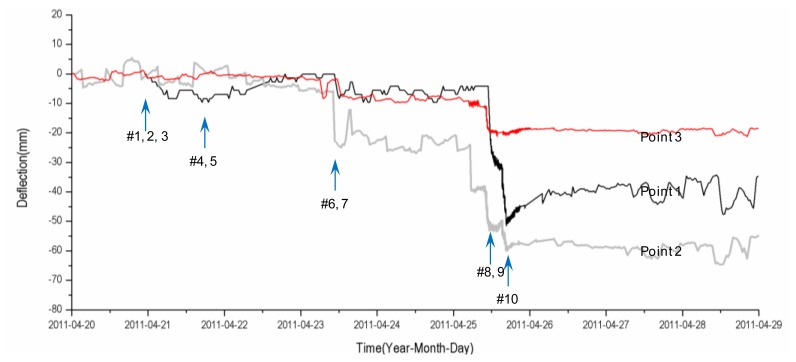
Vertical displacement measurements.

The long-term measurement results obtained during the construction period (from April 2011 to April 2012) show that the removal of the bents is the primary influence on the behavior of the structure. The locations of the bents and their removal sequence are shown in Figure 5, and Figure 7 shows the vertical displacements at the three measurement points (Figure 5) during a specific event, the removal of the bents in April 2011. The removal of bents 1, 2, and 3, which are located far from the measurement points, has a minimal effect on the behavior of the structure. However, the vertical displacements at the corresponding measurement points are substantial for the removal of the bents located closer to each measurement point. When bents 9 and 10, installed at the connecting points between the mega-trusses and the edge truss, are removed, large vertical displacements occur at measurement points 1 and 3 due to the behavior of the cantilever free end at the end of the mega-truss. The displacement at the two points reveals that point 1 incurs a greater displacement (52 mm) than point 3 (13 mm). This result can be explained by the fact that the free end of the mega-truss connected to point 1 is longer than the free end of the mega-truss connected to point 3 (Figure 5). In addition to the displacement caused by the behavior of the cantilever at the free end, the large vertical displacement at measurement point 2 is also caused by the deflection generated at the mid-span of the edge truss during the removal of bents 6 and 7, which are located at both ends of measurement point 2.

After the removal of the bents is completed, the largest vertical displacement (67 mm) among the three measurement points occurs at the center part (point 2) of the edge truss. This result is attributed to the addition of the displacement that occurs at the center region of the edge truss (as a long-span element) to the displacement of the free end of the mega-trusses (Point 1 and 3), which is triggered by the removal of the bents. A comparison of the vertical displacement for the building during construction measured using the LDS and the wireless sensor node and the calculated results of the structural analysis performed in the design phase of the building reveals that the measured values are lower than the calculated values, which verifies that safety evaluation during construction is feasible.

## 5. Conclusions

In this study, we developed a wireless displacement measurement system designed to use displacement measurements as a direct index for assessing the safety of and damage to a structure in SHM. The system was applied to a building under construction to verify the applicability of the proposed system.

The proposed measurement system consists of an LDS capable of performing long-range measurements and a customized sensor node to enable wireless communication. The sensor node consists of a sensor module, a CDMA communication module, a processor, and a power module. To ensure a reliable power supply, which is considered the most critical problem for field applications of wireless measurement systems, the power consumption during the non-measurement and non-transfer periods was minimized by establishing the measurement and transfer periods via the sleep/active modes. The timing of the active modes related to the measurement and transfer of data can be determined based on the measurement target, battery life, and mobile communication charges incurred by the CDMA, which enables suitable monitoring for various field situations via flexible management of the measurement and transfer periods.

The proposed wireless displacement measurement system was applied to a large-scale irregular building under construction to monitor the vertical displacements of the mega-truss elements during construction. The measured data from the LDS can be directly transferred to a remote monitoring server from the building site via CDMA communication, which overcomes the limitation of communication distance; thus, the measurement system installed onsite was configured with the LDS, and the wireless sensor node was connected by a short signal line.

The monitoring results indicated that a large vertical displacement occurred at the free end of the mega-truss, which was triggered by the removal of the temporary bents installed during the construction of the mega-trusses and the edge truss. In addition to the displacement of the free end of the mega-truss, the center component of the long-span edge truss also experienced large deflections, and consequently, the largest vertical displacement among the three installed wireless laser displacement measurement systems was measured at the center region of the edge truss. A comparison of the displacement values obtained from the pre-analysis performed in the design phase of the building and the obtained measurement values confirmed the validity of the proposed wireless displacement measurement system and the potential for safety evaluations of structural elements.
